# Testicular tissue cryopreservation and spermatogonial stem cell transplantation to restore fertility: from bench to bedside

**DOI:** 10.1186/scrt457

**Published:** 2014-05-28

**Authors:** Hooman Sadri-Ardekani, Anthony Atala

**Affiliations:** 1 Wake Forest Institute of Regenerative Medicine (WFIRM), Wake Forest School of Medicine, Winston-Salem, North Carolina 27101, USA; 2 Reproductive Biotechnology Research Center, Avicenna Research Institute, ACECR, Tehran 1177-19615, Iran; 3 Department of Urology, Wake Forest School of Medicine, Winston-Salem, North Carolina 27101, USA

## Abstract

Male infertility management has made significant progress during the past three decades, especially after the introduction of intracytoplasmic sperm injection in 1992. However, many boys and men still suffer from primary testicular failure due to acquired or genetic causes. New and novel treatments are needed to address these issues. Spermatogenesis originates from spermatogonial stem cells (SSCs) that reside in the testis. Many of these men lack SSCs or have lost SSCs over time as a result of specific medical conditions or toxic exposures. Loss of SSCs is critical in prepubertal boys who suffer from cancer and are going through gonadotoxic cancer treatments, as there is no option of sperm cryopresrvation due to sexual immaturity. The development of SSC transplantation in a mouse model to repopulate spermatozoa in depleted testes has opened new avenues of research in other animal models, including non-human primates. Recent advances in cryopreservation and *in vitro* propagation of human SSCs offer promise for human SSC autotransplantation in the near future. Ongoing research is focusing on safety and technical issues of human SSC autotransplantation. This is the time to counsel parents and boys at risk of infertility on the possibility of cryopreserving and banking a small amount of testis tissue for potential future use in SSC transplantation.

## Introduction

Male infertility is a problem in 7% of all men [1]. In 1696 sperm were first seen under the microscope and called ‘homunculi’ as it was believed that the sperm contained a miniature human [2]. Three centuries later, the development of intracytoplasmic sperm injection (ICSI) into an egg has revolutionized male infertility treatments as part of assisted reproductive technologies (ARTs) [3,4]. However, many men with primary testicular defects in sperm production due to genetic disorders or as a consequence of cancer treatments are still unable to become biological fathers. The identification of rat spermatogonial stem cells (SSCs) in 1971 as the foundation for spermatogenesis and sustaining male fertility [5] and the introduction of SSC transplantation in mice in 1994 opened new avenues for the field of male infertility treatments [6]. Since the discovery of the feasibility of SSC isolation and autotransplantation, it has been demonstrated in several species, including non-human primates [7]. Brian Hermann and colleagues [7] recently demonstrated successful autologous and allogeneic SSC transplantations in adult and prepubertal macaque testes that were previously rendered infertile with alkylating chemotherapy. As a result of these findings, translation of this technology to human studies is expected soon. This review focuses on several areas, including identifying patients that may benefit from testicular tissue banking to preserve SSCs, recent achievements in SSC technology, and concerns that need to be addressed before applying SSC autotransplantation in the clinical setting.

## Who may benefit from testicular tissue preservation and future SSC transplantation?

### Malignant diseases

**Figure 1:**
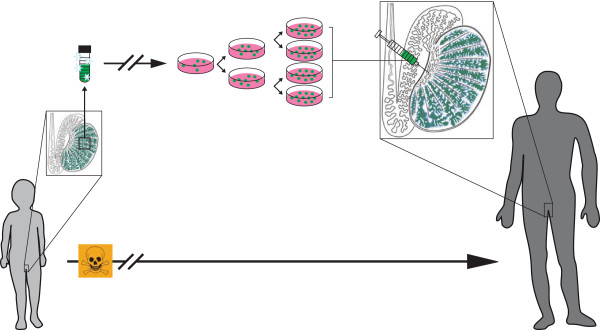
Schematic diagram showing testicular tissue cryopreservation and future spermatogonial stem cell autotransplantation to restore male fertility in high-risk patients.

Every year in the United States more than 12,000 children and adolescents aged under 20 years are diagnosed with cancer [8]. The overall cure rates of these cancer patients are approaching 80%; therefore, the number of childhood cancer survivors is increasing over time [8].It is known that either cancer [9] or cancer treatments [10] may adversely affect male reproduction. Chemotherapy and radiotherapy target rapidly dividing cells. These treatments not only eliminate malignant cells, but also affect germ cells. In the testis, spermatogonial cells divide rapidly and are very sensitive to cytotoxic agents, although the less active stem cells may also be killed [10]. Even in prepubescent boys, spermatogonial cells divide [11] and increase in number over time [12]. Thus, cancer treatments may result in temporary, long-term, or permanent gonadal failure in male cancer survivors [10]. In clinical practice, it is important to estimate infertility risk based on cancer type and cancer treatment protocols for each patient and consult with him and his parents (for prepubertal and adolescent patients) on his infertility risk (Tables 1 and 2) [13-15]. In adult men, semen cryopreservation before starting chemotherapy or radiotherapy is clinically approved as an efficient solution to preserve fertility by using ART procedures. Live births have been reported after insemination of stored sperm even after freezing for a period of 28 years [16]. In immature boys, spermatogenesis has not begun; therefore, storing testicular tissue prior to cancer treatments for future SSC autotransplantation could be an option (Figure 1).

**Table 1:** Estimation of infertility risk in different types of cancer

**High risk (>80%)**	**Intermediate risk (20-80%)**	**Low risk (<20%)**	**Very low/no risk**	**Unknown risk**
Any cancer requiring bone marrow transplant/stem cell transplant	Acute myeloblastic leukemia	Acute lymphoblastic leukemia	Thyroid cancer	Chronic myeloid leukemia
Brain tumor	Brain tumor	Germ cell tumors (without radiotherapy)		Colon cancer
Germ cell tumors	Hepatoblastoma	Nephroblastoma (without abdominal radiotherapy)		Gastrointestinal stromal tumor
Hodgkin lymphoma	Hodgkin lymphoma	Retinoblastoma		Head and neck cancer
Neuroblastoma	Neuroblastoma	Testicular cancer		Non-small cell lung cancer
Nephroblastoma	Non-Hodgkin lymphoma	Wilms’ tumor		Pancreatic cancer
Non-Hodgkin lymphoma	Sarcoma			
Sarcoma	Testicular cancer			
Testicular cancer	Wilms’ tumor			

**Table 2:** Estimation of infertility risk using different types of cytotoxic treatments

**High risk (>80%)**	**Intermediate risk (20-80%)**	**Low risk (<20%)**	**Very low/no risk**
Any alkylating agent plus total body irradiation, pelvic radiation, or testicular radiation (for example, procarbazine, nitrogen mustard, cyclophosphamide)	BEP × 2–4 cycles (bleomycin, etoposide, cisplatin)	ABVD (doxorubicin, bleomycin, vinblastine, dacarbazine)	Radioactive iodine
Busulfan (≥600 mg/m^2^)	Carboplatin cumulative dose ≤2 g/m^2^	CHOP (cyclophosphamide, doxorubicin, vincristine, prednisone)	Testicular radiation dose (<0.2 Gy)
Busulfan/cyclophosphamide	Cisplatin cumulative dose <400 mg/m^2^	COP (cyclophosphamide, vincristine, prednisone)	
CBV (cyclophosphamide, BCNU, etoposide), BCNU cumulative dose ≥300 mg/m^2^	Testicular radiation dose (scatter from abdominal/pelvic radiation) (1–6 Gy)	NOVP (mitoxantrone, vincristine, vinblastine, prednisone)	
ChIVPP (chlorambucil, vinblastine, prednisone, procarbazine)		OEPA × 2 cycles (vincristine, etoposide, prednisone, doxorubicin)	
ChIVPP/EVA (chlorambucil, vinblastine, prednisone, procarbazine, doxorubicin, vincristine, etoposide)		Testicular radiation dose (0.2-0.7 Gy)	
COPP × 6 cycles (cyclophosphamide, vincristine, procarbazine, prednisone)			
COPP/ABVD (cyclophosphamide, vincristine, procarabazine, prednisone, doxorubicin, bleomycin, vinblastine, dacarbazine)			
Cranial/brain radiation ≥40 Gy			
Cyclophosphamide >7.5 g/m^2^			
Cyclophosphamide as bone marrow transplant conditioning			
Cyclophosphamide (19 g/m^2^) plus total body irradiation			
MOPP > 3 cycles (nitrogen mustard, vincristine, procarabazine, prednisone)			
MOPP/ABVD (nitrogen mustard, vincristine, procarabazine, prednisone, doxorubicin, bleomycin, vinblastine, dacarbazine)			
MVPP (nitrogen mustard, vinblastine, prednisone, procarabzine)			
Procarbazine cumulative dose ≥4 g/m^2^			
Testicular radiation dose >2.5 Gy in adults			
Testicular radiation dose ≥6 Gy in children			
Total body irradiation			

### Non-malignant diseases need cytotoxic treatments

In addition to malignant diseases, certain benign hematological disorders, such as myelodysplasia, sickle cell disease, aplastic anemia, thalassemia major, and Fanconi anemia, and severe autoimmune diseases unresponsive to immunosuppressive therapy, such as juvenile idiopathic arthritis, juvenile systemic lupus erythematosus, systemic sclerosis and immune cytopenias, necessitate administration of high dose chemotherapy [17-19]. This often leads to severe, dose-dependent and sometimes irreversible spermatogenic damage [20]. Dependent on treatment types (Table 2), these patients may also need to be counseled for fertility preservation.

### Klinefelter syndrome

Klinefelter syndrome (KS; 47,XXY) is a progressive testicular failure causing small firm testes, androgen deficiency, and azoospermia [21]. This syndrome has been reported in 1 out of 660 live male births [22] and represents approximately 15% of azoospermia in infertile men [23]. KS cases have normal sexual hormones during childhood and initiate puberty at the same age as normal children; however, around mid-puberty the testes begin to deteriorate with the loss of germ cells [24]. Successful testicular sperm extraction is expected in half of KS patients [24]; a recent study showed 70% success for microscopic testicular sperm extraction in 10 cases where the males were aged between 14 and 22 years [25]. Preserving testicular tissue containing SSCs before puberty may help some KS boys in the future [26]. Less than 10% of KS is diagnosed before puberty [22]; therefore, a cost-effective and easy method (for example, PCR) to screen these children before puberty is needed.

### Cryptorchidism

Failure in congenital testicular descent - cryptorchidism - is the most frequent genital abnormality, affecting approximately 1% of mature births [27]. In a study of 89 cryptorchid boys who underwent bilateral testis biopsy during orchiopexy operation, 70% of scrotal testes had an impaired transformation of A_dark_ spermatogonia, indicating that cryptorchidism is a bilateral disease [28]. Up to 20% of boys with unilateral cryptorchidism experience fertility problems and this figure increases up to 70% for boys with bilateral cryptorchidism [29]. Paternity rate decreases significantly in corrected bilateral cryptorchidism (65%) compared with unilateral cryptorchidism (89.7%) and control men (93.2%) [30]. Because of gradual diminishing germ cell number in these patients, it may be an option to store a portion of the testis biopsy, which can be harvested during the orchiopexy operation [31,32].

## Testicular tissue biopsy and cryopreservation

**Figure 2:**
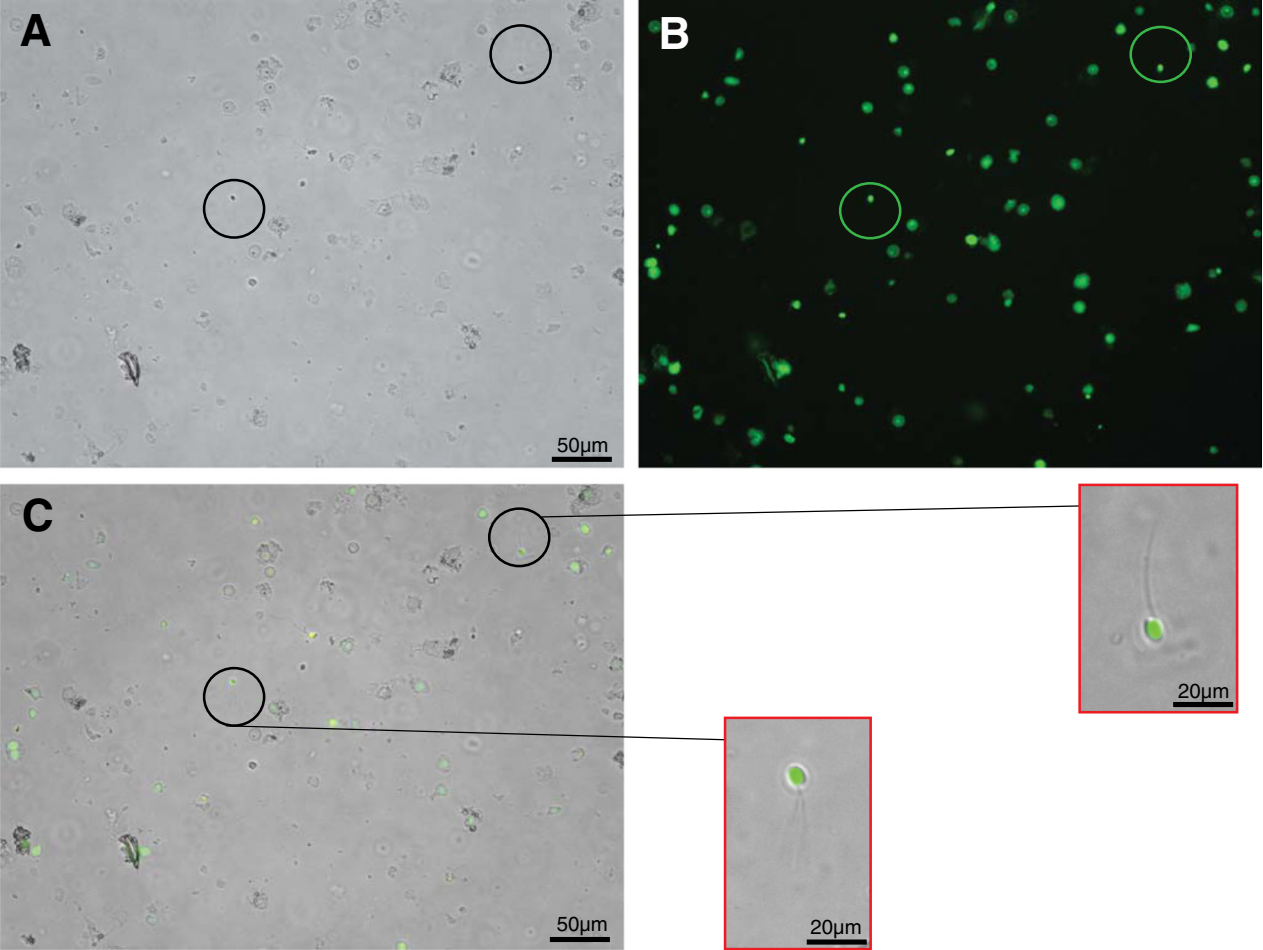
**Testicular sperm viability after freeze and thaw.** Live sperms were stained with Calcein (green fluorescent). **(A)** Bright field; **(B)** fluorescent; **(C)** merged. A LIVE/DEAD Sperm Viability Kit (L-7011 Invitrogen, Life Technologies Ltd, Paisley, UK) was used for staining thawed testicular sperms. Testicular tissue for research was obtained from transplant donors through the National Disease Research Interchange.

Testicular biopsy is an open surgical procedure and needs to be performed under general anesthesia in children. Retrieving tissue from only one testis is suggested to minimize manipulation [14] and the size of tissue may vary between 80 and 250 mm^3^ based on testicular size in the different age groups [33]. To minimize the risk of general anesthesia, this process should be performed at the same time as other clinical procedures (for example, bone marrow biopsy, central line replacement or orchiopexy) when possible. Based on our [34] and other groups’ experiences [14,35] no major surgical complications occurred during or after testicular biopsy. Long-term follow-up of cryptorchid boys who had undergone testicular biopsy during orchiopexy showed no negative effects such as producing anti-sperm antibody or testicular scars [32]. Onset of sperm production (spermarche) is an early pubertal event. The median age of spermarche is estimated to be around 13 to 14 years, with a range between 11 and 17 years [36,37]. Thus, we recommend searching for testicular sperm in specimens from all boys aged 10 years or older, since freezing testicular sperm in glycerol-based medium [38] for use in ICSI is available in most ART laboratories [39]. Protocols for freezing small samples (2 to 4 mm^3^) of immature human testicular tissue using dimethyl sulfoxide (DMSO) as the main cryoprotectant showed good structural integrity of testicular tubules, and pre-tubular and intra-tubular cells after thawing [11,40]. Xenotransplantation of cultured DMSO frozen SSCs from immature human testes showed the migration ability of SSCs to the base membrane of mouse seminiferous tubules without differentiation to mature germ cells [34]. The same cryopreservation method has been used for storing mice SSCs for longer than 14 years. Fertile offspring were derived after transplanting these long-term stored SSCs [41]. Our current testicular tissue banking protocol at Wake Forest Baptist Health for boys at risk of infertility indicates that, if testicular sperm are found, then half of the tissue will be frozen (Figure 2) in routinely used egg yolk-glycerol-based cryopreservation medium to preserve sperms for ICSI and the other half will be frozen to preserve the SSCs in DMSO-based medium for future culture and transplantation.

## Isolation and *in vitro* propagation of spermatogonial stem cells

### Spermatogonial stem cell isolation

The first successful isolation of human SSCs was reported from six infertile adult men in 2002 [42]. In that study, isolated human SSCs were able to colonize and survive for 6 months in mice recipient testes even after a freeze-thaw procedure. Numbers of colonized human SSCs in mouse seminiferous tubules were evaluated up to 6 months after transplantation. Observation of clusters of human SSCs about 1 month after transplantation suggested the proliferation of these cells in mouse testes. Human cells remained up to 6 months in mouse testes, although their numbers significantly decreased by 2 months after transplantation. No meiotic differentiation of human germ cells in mouse testes was observed [42]. Recently, in a study of prepubertal boys diagnosed with cancer, SSCs were isolated and demonstrated stem cell activity after xenotransplantation to mouse testes similar to that seen in human adult SSCs [43]. This study used biopsies from nine boys aged 2 to 10 years and a preliminary estimation indicated that spermatogonial cells comprised about 3% of the cell population from these biopsies [43]. The number of SSCs in the testis is very low. In mouse testis only 0.03% of germ cells and 1.25% of spermatogonial cells are estimated to be stem cells [44,45]. In contrast to rodents, human spermatogonial cells can be divided into two subgroups, A_pale_ and A_dark_, according to their nuclear staining with hematoxylin after Bouin’s fixation [46]. A_dark_ spermatogonia in normal circumstances are quiescent cells and are thought to be reserve (stem) cells [45]. Current SSC isolation methods are based on two-step enzymatic digestion [47]. Investigators have enriched human spermatogonial cells using magnetic activated cell sorting (MACS) with markers such as GFRA1^+^[48], GPR125^+^[49], SSEA4^+^[50], and HLA-ABC^−^/CD9^+^[51] or using fluorescence-activated cell sorting (FACS) by isolating EpCAM^+^/HLA-ABC^−^/CD49e^−^ cells [52]. Ideally, isolation of pure SSCs is expected, but no specific marker has been found to identify the stem cells in testis [53]. Finding suitable marker(s) is a formidable task [54].

### *In vitro* propagation

**Figure 3:**
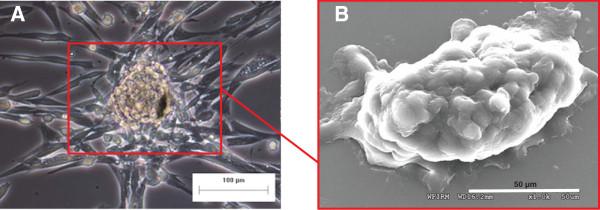
**Germ line stem cells cluster in human testicular cell culture.** The presence of these germ line clusters has been described previously [34,62]. **(A)** Bright field; **(B)** scanning electron microscopy. Testicular tissue for research was obtained from transplant donors through the National Disease Research Interchange.

In immature boys, the size of the testis is small with a rare population of SSCs; therefore, isolation of these cells from a small testicular biopsy yields a very limited number of stem cells. Based on animal studies, SSC transplantation efficiency depends on the number of transplanted SSCs, with an almost linear correlation [55]. Therefore, increasing the number of SSCs *in vitro* is necessary before transplantation. Successful *in vitro* culturing of SSCs has been reported in several species, including mouse [56,57], rat [58], bovine [59], hamster [60], and dog [61]. Recently, *in vitro* propagation of human SSCs from both adult [62] and prepubertal [34] testes was established. In these systems, human SSCs are supported by a feeder layer from the same patients’ testicular somatic cells. Germ line stem cell clusters formed within 2 to 4 weeks of culture (Figure 3). Xenotransplantation of human testicular cells from different time points of *in vitro* culture into nude mice testes showed that human SSCs could be maintained *in vitro* for more than 15 weeks with a doubling time of 3 to 7 days [34,62]. Optimization of this culture system based on US Food and Drug Administration regulations and current good tissue practice requirements are imperative before use in a clinical application.

## Safety and technical issues of spermatogonial stem cell transplantation

### Genetics and epigenetics stability

Harvesting testicular tissue via biopsy, freezing and thawing the tissue, and cell isolation and culturing are all processes that may affect the integrity of SSCs. Alterations in manipulated cells may occur in the genome, in the epigenome, or in both [63-65]. There are reports that show the genetic stability of other stem cell populations during *in vitro* culture [66,67]. Since SSCs are the cells that transmit genetic information to the next generations, concerns about SSC stability are much more important than those about somatic cells. A study on transplantation of isolated SSCs from C57Bl/WBRe donor mouse (without culturing) to the testes of W/Wv-mice [68] showed normal development (length and weight) compared with controls for first and second generation offspring. DNA extracted from post-transplantation spermatozoa, liver, kidney and placenta revealed no differences in methylation patterns of genes for Igf2, Peg1 and a-Actin between offspring of transplanted and control mice [68]. Kanatsu-Shinohara and colleagues [69] showed that *in vitro* expansion of mouse SSCs over 24 months continued with normal karyotype and stable androgenetic imprinting. The offspring of recipient mice were fertile and also had a normal imprinting pattern. However, genetic alterations or epigenetic patterns of isolated and cultured human SSCs have not yet been determined.

### Contamination with cancer cells

The most important concern regarding SSC autotransplantation is the risk of reintroducing malignant cells to the cancer survivor. This is very important in non-solid hematopoietic cancers, as malignant cells can migrate through the blood circulation and infiltrate the testis [70]. It has been demonstrated that intraluminal injection of as few as 20 leukemia cells into the testes of recipient rats could induce disease relapse in three out of five animals [71]. A few studies have tried to eliminate malignant cells from mouse, non-human primate and human testicular cell suspensions [52,72-75]. These studies used different surface markers for MACS or FACS of contaminating cells. Currently there is no specific marker for purifying SSCs [76] and these cells share several biomarkers with other stem cells and cancer cells, especially hematopoietic cells [77]; therefore, the sorting methods have not yielded tumor cell-free populations. The most recent study attempting to remove human leukemia cells from testicular cells using the markers EpCAM^+^/HLA-ABC^−^/CD49e^−^ showed some progress [52]; however, the bioassay method used for post-sorting detection of leukemia cells was not sensitive enough (0.2% sensitivity) and the false negative rate was high (>60%) [52,75]. Using other detection methods with higher sensitivity, such as minimal residual disease PCR (up to 0.0001% sensitivity) [78] or tumor cell imaging (to detect as few as 3 to 10 cells) [79] are recommended. Our recently published pilot study using minimal residual disease PCR to track leukemia cells in a human SSC *in vitro* propagation system showed leukemia cells were eliminated after 26 days of co-culturing with spermatogonial cells [80].

### Spermatogonial stem cell injection

A mouse model for injection of SSCs into the testis is possible with the microinjection of the SSCs into the seminiferous tubules, into the rete testis, or into the efferent duct [81]. However, in larger animals like bovine, monkey and even human cadaver, studies have shown that injection of SSCs into the seminiferous tubules or the efferent duct was not successful [82]. This is because of high resistance of the lamina propria and coiled seminiferous tubules in larger animals. The most promising models for SSC injection into human testis is ultrasound-guided injection into the rete testis [82,83]. In the most recent study on autopsied human testes, injecting 8 to 16 million cells in a volume of 800 to 1,400 μl via a 23 gauge needle could fill up to 40% of the seminiferous tubules in 1 to 2 minutes [83]. There is only one reported clinical trial of SSC autotransplantation, in seven cancer survivors [84], but the details of this study and patient follow-up data have not been published. Further investigation is necessary to optimize the injection procedure as well as compare ultrasound-guided versus open surgery for SSC transplantation.

## Points of view of patients and their families

It is important to understand how patients and their families feel about fertility preservation and testicular tissue banking. Psychosocial studies clearly demonstrate a high incidence of negative reactions to infertility and its negative effect on overall life satisfaction and well-being [85]. The main target groups for testicular tissue cryopreservation for future SSC autotransplantation are children, which presents difficulties for discussion of future reproduction and family planning. Childhood cancer survivors who transit into adulthood express concerns about fertility and fathering children [86]. At least half of the parents of boys who suffer from cancer agree with performing testicular biopsy to preserve SSCs [35,87,88]. Parents choose fertility preservation even if the chance of infertility is low (≤20%) and the success rate of future SSCs transplantation will also be low (≤20%) [88]; these findings show the great importance of fertility preservation for families.

## Follow-up after spermatogonial stem cell transplantation

Both childhood and adult cancer survivors are interested in understanding the risks of passing on genetic damage capable of causing adverse outcomes in their children [89]. Reviewing a cohort of 8,670 children born between 1994 and 2004 with a paternal history of cancer versus 17,690,795 children without a paternal history of cancer showed a higher incidence of major congenital abnormalities in the offspring of male cancer survivors (3.7 out of 100) than in those of fathers with no history of cancer (3.2 out of 100) [90]. Around 5% of children (508 out of 8,670) were conceived using ART, either *in vitro* fertilization (5%) or ICSI (95%), with the higher risk of abnormalities with *in vitro* fertilization (two times more) compared to ICSI or natural conception [90]. Previous studies have shown no significant differences in the outcome of pregnancy in cancer survivors [91,92]. However, ICSI (when it is required) and prenatal diagnosis tests (for example, amniocentesis) during pregnancy in cancer survivors may overcome this small risk.

As clinical trials of SSC autotransplantation are initiated, it is necessary to monitor the health of recipient men. Like other ART treatments, pregnancy and the offspring should be followed for any major abnormalities.

## Conclusion

SSCs are germ line stem cells that reside in the basement membrane of the seminiferous tubule in the testis. They are the foundation of spermatogenesis for the production of sperm after puberty. In 1994 Brinster’s group [6] reported a SSC assay in mice that shows the ability of these cells to generate a colony of spermatogenesis after transplantation in the seminiferous tubules of a recipient male. Since then, researchers in the field of male infertility have searched for new clinical tools to help more men who suffer from primary testicular failure. Immature boys at risk of losing their SSCs, mostly cancer patients, are the main target group that may benefit from testicular tissue cryopreservation and SSC autotransplantation. Progress in the field of SSC transplantation in animal studies, including non-human primates, has been shown. Effective freezing methods for adult and prepubertal testicular tissue are available and recently *in vitro* propagation of human SSCs with the ability to colonize the basement membrane of testes has been established. Therefore, translation of SSC autotransplantation to humans is expected to be possible in the near future. The families of prepubertal and adolescent male patients are eagerly awaiting fertility preservation by means of testicular tissue banking and utilization in future clinical applications. Simultaneous to the ongoing research on safety and technical issues of human SSC autotransplantation, it is necessary to counsel parents and the boys at risk of infertility on the possibility of cryopreserving a small testis biopsy in experimental SSC banking.

## Note

This article is part of a thematic series on *Stem cells in genitourinary regeneration* edited by John Jackson. Other articles in the series can be found online at http://stemcellres.com/series/genitourinary

## Abbreviations

ART: Assisted reproductive technology; DMSO: Dimethyl sulfoxide; FACS: Fluorescence-activated cell sorting; ICSI: Intracytoplasmic sperm injection; KS: Klinefelter syndrome; MACS: Magnetic activated cell sorting; PCR: Polymerase chain reaction; SSC: Spermatogonial stem cell.

## Competing interests

The authors declare that they have no competing interests.
